# Correlation of microbiomes in “plant-insect-soil” ecosystem

**DOI:** 10.3389/fmicb.2023.1088532

**Published:** 2023-01-30

**Authors:** Guomeng Li, Peng Liu, Jihan Zhao, Liangyinan Su, Mengyu Zhao, Zhengjie Jiang, Yang Zhao, Xiping Yang

**Affiliations:** ^1^State Key Laboratory of Conservation and Utilization of Subtropical Agro-Bioresources, Guangxi Key Laboratory of Sugarcane Biology, Guangxi University, Nanning, China; ^2^Key Laboratory of Crop Cultivation and Tillage, College of Agriculture, Guangxi University, Nanning, China

**Keywords:** microbiome, sugarcane stem, striped borer, soil, resistance

## Abstract

**Introduction:**

Traditional chemical control methods pose a damaging effect on farmland ecology, and their long-term use has led to the development of pest resistance.

**Methods:**

Here, we analyzed the correlations and differences in the microbiome present in the plant and soil of sugarcane cultivars exhibiting different insect resistance to investigate the role played by microbiome in crop insect resistance. We evaluated the microbiome of stems, topsoil, rhizosphere soil, and striped borers obtained from infested stems, as well as soil chemical parameters.

**Results and Discussion:**

Results showed that microbiome diversity was higher in stems of insect-resistant plants, and contrast, lower in the soil of resistant plants, with fungi being more pronounced than bacteria. The microbiome in plant stems was almost entirely derived from the soil. The microbiome of insect-susceptible plants and surrounding soil tended to change towards that of insect-resistant plants after insect damage. Insects’ microbiome was mainly derived from plant stems and partly from the soil. Available potassium showed an extremely significant correlation with soil microbiome. This study validated the role played by the microbiome ecology of plant–soil-insect system in insect resistance and provided a pre-theoretical basis for crop resistance control.

## 1. Introduction

As society became increasingly concerned about sustainable agriculture, the soil, on which plants and micro-organisms depend, should be better managed to regulate the structure of the soil microbiota to provide a contribution to plant growth and resistance (Reinhold-Hurek and Hurek, 2011; Delgado-Baquerizo et al., 2016, 2018). Sugarcane is a major source of sugar and bioethanol (Li and Yang, 2015). The sugarcane stem borer is one of the most common and serious pests of sugarcane and is one of the key factors in the reduction of sugarcane yields (Wang et al., 2018). In the early stages of sugarcane growth, the striped borers infest the stem base of sugarcane seedlings, causing dead heart seedlings (Li et al., 2019). In the middle and late stages of sugarcane growth, the striped borers infest the stems of sugarcane, causing stem breakage and a decrease in sucrose content (Showler, 2016). The infestation can therefore last for the entire planting period (Cristofoletti et al., 2018). Current control measures against the stem borer are mainly systemic insecticides sown at the seedling stage, which are conditionally effective, but in the middle and late stages of growth, when the stem is infested with the borer, spraying against the insects is not as so effective as expected (Mehnaz, 2013). Over time, the stem borer could also develop a degree of resistance to insecticides. Therefore, the green control of the stem borer has received a lot of attention from the community (Gao et al., 2016; Cristofoletti et al., 2018). At present, researchers engaged in microbiome control have screened out parasitic microorganisms, such as *Pseudomonas aeruginosa*, that had significant killing power against the pest for control purposes (Chen et al., 2012; Cristofoletti et al., 2018; de Mello et al., 2020). However, the activity of microbiome pesticides varies from region to region due to several factors such as geography. At the same time, some researchers hope to identify useful microorganisms and their secondary metabolites from the soil with anti-pest effects, and use them for pest control (Lacey et al., 2015; Mascarin et al., 2019; San-Blas et al., 2019; Zhang et al., 2019; Franco et al., 2022). Therefore, it is highly desirable to identify functional microorganisms and reveal the interaction between microbiota composition and plant-insect resistance (Mehnaz, 2013).

It was well known that the soil is extremely rich in microbiota bacteria and fungi. Plants also harbor symbiotic microbiota that is important for their development and response to the environment (Delgado-Baquerizo et al., 2016, 2018). When plants take root in the soil, a proportion of the soil microbiota could transfer and settle in the plant, and form a dynamic relationship with the plant during its growth and development (Agrawal et al., 2018; Hassani et al., 2018). The above-ground parts of plants, such as stems, harbor specific commensal, parasitic or pathogenic bacteria, and fungi, at least partly from the soil (Bai et al., 2015; Cortois et al., 2016). The inter-root soil contains a variety of microorganisms that are beneficial to plant growth and health, such as nitrogen-fixing bacteria, photosynthetic bacteria, and microorganisms that help improve resistance to adversity (Almario et al., 2017; Durán et al., 2018; Etalo et al., 2018; Hou et al., 2021; Wu et al., 2022). Variations in these microbiotas play an great role in the growth and development of plants. However, little was known about their relationship with plant resistance to insects (Khan et al., 2022). Numerous factors influence how plant-associated microorganisms generally affect the fitness and health of hosts(de Faria et al., 2021; King et al., 2022), including the genotype of the host and microbes, interactions within the microbiota, and a variety of abiotic factors. Insect survival is also associated with a wide range of microorganisms (Borgström et al., 2017; Agrawal et al., 2018). These microbes could act as a disease-causing pathogen or playing a role in insect defense, detoxification, or digestion of food (Chen et al., 2016; Li et al., 2022). Microorganisms of herbivorous insects have also been found to be present in plants. Through plants, the soil microbiota could be incorporated into the microbiota of insects (Frago et al., 2012; Heinen et al., 2018; French et al., 2021). Other studies have shown that in addition to directly ingesting certain symbiotic bacterial and fungal microbiota from the soil, herbivorous insects can also do so from the environment (Koricheva et al., 2009). Therefore, changes in the soil microbiota might also lead to changes in the insect microbiota, thereby altering insect performance through the plant’s microbiota or through the direct interaction between soil and insects (Borgström et al., 2017; Hannula et al., 2019; Markalanda et al., 2022).

As studies on the microbial ecology of plant environments become more advanced, a comprehensive and systematic study of the plant–soil microecological environment should be carried out based on high-throughput sequencing data. This study aimed to investigate the correlation between plant insect resistance traits and response to herbivores with the microbial ecology in which they are found, through a comprehensive evaluation of bacterial and fungal microbiomes and soil chemistry, and to preliminarily screen for microbiota and microbial functions that are significantly correlated, and to understand fully the essential contribution of environmental microbial ecology to insect resistance traits in plants.

## 2. Materials and methods

### 2.1. Sampling of plant materials, insects, and soil

The experimental samples were divided into three groups: healthy borer-resistant variety [GT 22 (a new generation of main sugarcane variety in Guangxi)], healthy borer-susceptible variety [GT 42 (a new generation of main sugarcane variety in Guangxi)] and infested borer-susceptible variety (GT 42). Each group had three sampling sections: stem, top soil, and rhizosphere soil. For borer-infested plants, we also collected the corresponding striped borers. The insect sample was *Chilo sacchariphagus Bojer* (Bleszynski, 1970). Lepidoptera. Aphididae. The morphological judgement of insects was based on *MANAGEMENT OF SUGARCANE DISEASES AND PESTS* (Huang et al., 2014). The larvae are 15 mm long, yellowish-white, with 4 purple longitudinal lines on the dorsal surface (2 on the subdorsal line and 2 on the upper line of the valve); each node has black trichomes, and the dorsal surface of the ventral node has large dark brown trichomes arranged in squares in the center. The striped borer was selected from the larval stage. Three biological replicates were set up for each group of sampling. GT 22 and GT 42 varieties were planted and sampled in experimental plots under the same tillage conditions at Guangxi University experimental field, Fusui County, Chongzuo City, Guangxi Zhuang Autonomous Region. Samples were collected in June 2021, test field coordinates: 22° 50´ N, 107° 77′ E.

Plant stems were cut off, surface sterilized with 75% alcohol. The inner stems were used as plant samples or subsequent endophytes extraction. Top soil samples were taken from the two to 10 cm surface soil areas of the selected plant materials. Rhizosphere soil was collected by carefully digging out the roots, shaking off large pieces of loose soil, gently brushing them down with a clean brush. Soil samples were passed through a 2 mm sterilized mesh sieve to remove residual plant root residues. Insect samples were surface sterilized by repeatedly soaked three times in 75% alcohol and washed with sterile water. All samples were snap-frozen at −80°C. The description of the experiment was collected in groups as shown in Table 1.

**Table 1:** Design and samples of the experiment.

**Sampling site**	**GT 22**	**GT 42**
	**Health**	**Insect pests**
Stem	H2201S	H4201S	I4201S
	H2202S	H4202S	I4202S
	H2203S	H4203S	I4203S
Topsoil	H2201T	H4201T	I4201T
(5–15 cm)	H2202T	H4202T	I4202T
	H2203T	H4203T	I4203T
Rhizosphere soil	H2201R	H4201R	I4201R
(25–35 cm)	H2202R	H4202R	I4202R
	H2203R	H4203R	I4203R
Borers	B1	B2	B3

### 2.2. Sample DNA extraction

Soil DNA were extracted using TIANamp Soil DNA Kit (Spin Column). Insect sample DNA was extracted using TIANamp Genomic DNA Kit (Spin Column). Bacterial sequencing region was 799F_1193R, and fungal sequencing region was ITS1F_ITS2R. Amplification primers targeting the sequencing regions were designed for testing the extracted DNA (Supplementary Table S3). The PCR reaction system and reaction conditions were shown in Supplementary Table S4. All samples were prepared in triplicate. The PCR products were extracted from 2% agarose gels and quantified using the AxyPrep DNA Gel Extraction Kit (Axygen Biosciences, Union City, CA, United States), and performs purification using a Quantus™ fluorometer (Promega, USA), according to the manufacturer’s instructions.

### 2.3. Soil physicochemical properties determination

Determination of soil physicochemical properties refer to “Soil Agrochemical Analysis” (3rd edition), by Bao (2000, Reprint). The pH value was determined by potentiometric method, organic carbon by potassium dichromate external heating method, total nitrogen by Kjeldahl method, total phosphorus by NaOH fusion-molybdenum antimony anti-colorimetric method, total potassium by NaOH fusion, flame photometric method, easily oxidized organic carbon by potassium permanganate oxidation method, available potassium by NH4DAc leaching, flame photometric method.

### 2.4. Bioinformatics and statistical analysis

Purified amplicons were pooled in equimolar amounts, and paired-end sequencing was performed on the Illumina MiSeq PE300 platform/NovaSeq PE250 platform (Illumina, San Diego, USA). according to the standard protocol of Majorbio Bio-Pharm Technology Co. Following demultiplexing, the resulting sequences were quality filtered with fastp (0.19.6) (Chen et al., 2016) and merged with FLASH (V1.2.11) (Magoč and Steven 2011). High-quality sequences were then denoised using the DADA2 (Callahan et al., 2016) plugin in the Qiime2 (Bolyen et al., 2019) pipeline, which obtains single-nucleotide resolution based on in-sample error profiles.DADA2 removes annotated chloroplast and mitochondrial sequences from all samples. All sample sequence numbers were drawn flat at the minimum sample sequence number (bacteria: I4201, 16,306. Fungi: H4203S, 32,272). Species taxonomy analysis of ASVs was based on bacteria: silva138/16s_bacteria/fungi: unite8.0/its fungi, using the Naive Bayes classifier in Qiime2, with a confidence level of 0.7. The raw Illumina reads for this study can be found at NCBI Sequence Read Archive database with accession number PRJNA845813, PRJNA845815, PRJNA845818, PRJNA845819, PRJNA845829, PRJNA845823.

In the analysis, we selected the intersection microbiome of three biological replicates as the analyzed data. Community composition analysis and correlation analysis were performed using the feature communities. For the difference comparisons, we performed one-way ANOVA tests using IBM SPSS Statistics 25 software. Parameters were chosen: LSD, Duncan (D) and chi-square test (H). Part of the data analysis was performed on the Meguiar’s BioCloud platform^1^ and the other part of the data analysis was performed in R (4.1.2) and Tutools^2^ were used for the mapping part.

## 3. Results

### 3.1. Differences in microbiomes between eco-systems of insect resistant and susceptible sugarcane varieties

**Figure 1:**
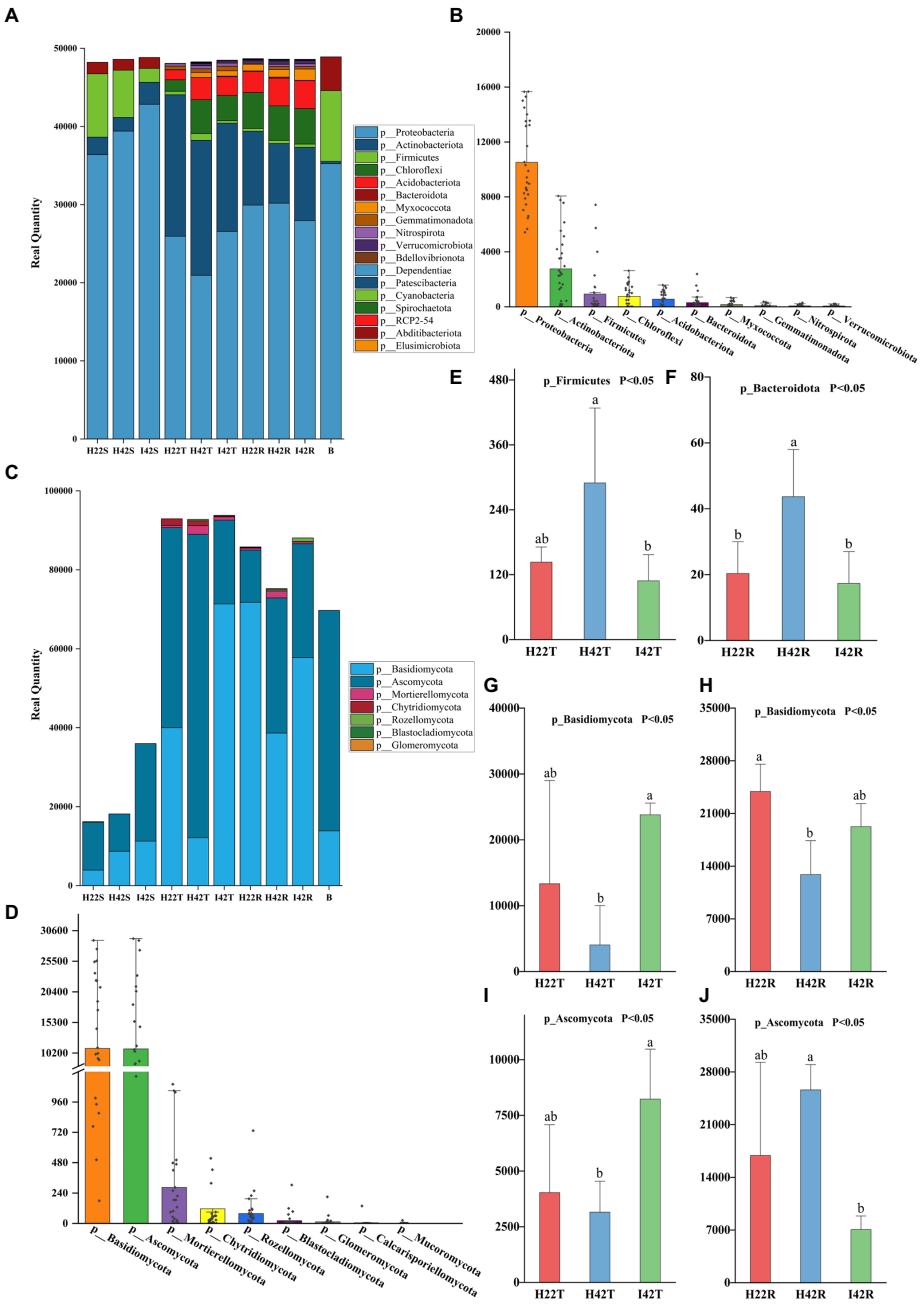
Composition of microbiota phylum levels. **(A)** Histogram of species stacking at the bacterial phylum level for experimental samples. The figure shows the full phylum-level microbiome obtained from the annotation, with the number percentages indicated by the length of the bars in the figure. **(B)** Histogram of the percentage of major species at the phylum level of the bacterial microbiome. The figure shows the distribution of individual samples at the major phylum level microbiomes, arranged in descending order of total sample species abundance. **(C)** Histogram of species stacking at the fungal phylum level for experimental samples. **(D)** Histogram of the percentage of major species at the phylum level of the fungal microbiome. **(E-J)** The gate-level microbiome that showed the expected significant level of difference in resistance associated with the different experimental subgroups. Error bars indicate standard deviation (+/–SD) and letters indicate differences between each subgroup. Differences were significant at the *p* ≤ 0.05 level calculated using the chi-squared test (H).

**Figure 2:**
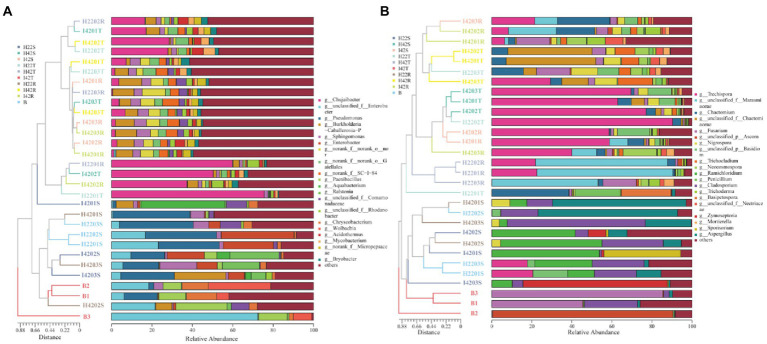
Figure of horizontal abundance of microbiome genera, with clustering trees drawn by bray weighting algorithm on the left and icons in descending order of richness on the right. **(A)** Genus level abundance map of bacterial microbiota. **(B)** Genus level abundance map of fungal microbiota.

To investigate the relationship between microbiome, and sugarcane resistance to the striped borer, we analyzed the bacterial and fungal communities of stems, topsoil, and rhizosphere soils of insect-resistant and insect-susceptible sugarcane varieties. We found the microbiome community composition of the stems differed between insect-resistant and insect-susceptible varieties at the phylum and genus level (one-way ANOVA tests, *p* < 0.05). A total of four phylum-level bacterial microbiota were obtained in sugarcane stems, including *Bacteroidota*, *Firmicutes*, *Actinobacteriota* and *Proteobacteria*. The microbiome species of the stems of insect-resistant and insect-susceptible plants were the same, but we found significantly higher relative abundances (RAs) of *Actinobacteriota* in insect-resistant plants than in insect-susceptible varieties (*p* < 0.05). At the genus level, we obtained a total of 26 bacterial microbiota, of which 10 genera were common to insect-resistant sugarcane plants, nine unique to insect-resistant plants and seven unique to insect-susceptible plants. We found *Enterobacteriaceae* was significantly higher in insect-resistant plants than in insect-susceptible plants (*p* < 0.05). And *Escherichia-Shigella* 0accounted for the highest percentage of the unique flora of insect-resistant plants (Ras = 1.29%, here the relative abundance percentages were calculated by taking the average of the samples, the following RAs percentages appear to be calculated in the same way.). In sugarcane stems, three phyla of fungal microbiota were obtained: *Ascomycota*, *Rozellomycota* and *Basidiomycota*. Insect-resistant sugarcane plants contained fungi of all three phyla, whereas insect-susceptible plants did not contain *Rozellomycota*. At the genus level, we obtained 11 fungal microbiomes, of which three genera were common to the resistant plants, six unique to the insect-resistant plants and two unique to the insect-susceptible plants. *Trechispora* was the most represented group unique to the insect-resistant plants (Ras = 5.87%), whereas *Nigrospora* was the most abundant microflora unique to the insect-susceptible plants (Ras = 1.99%) (Figures 1, 2).

Also, the microbiome community composition of the topsoil differed between the insect resistant and susceptible varieties at the phylum and genus level (one-way ANOVA tests, *p* < 0.05). A total of nine phylum level bacterial microbiota were obtained in the topsoil of insect resistant and susceptible plants. A total of 104 bacterial microbiota were obtained at the genus level, of which 54 were common to topsoil of insect-resistant and insect-susceptible plants, five unique to topsoil of insect-resistant plants and 45 unique to topsoil of insect-susceptible plants. *Ramlibacter* was the most abundant microbiome unique to the topsoil of resistant plants (Ras = 0.11%). *norank_f__norank_o__norank_c__AD3* was the most abundant microbiome unique to the topsoil of susceptible plants (Ras = 0.77%). Then, we found four fungal microbiome groups, *Ascomycota*, *Mortierellomycota*, *Basidiomycota* and *Chytridiomycota* in both topsoil of resistant and susceptible plants. At the genus level, we obtained a total of 39 fungal genera, of which 21 were common, three unique to topsoil of insect-resistant plants and 15 unique to topsoil of insect-susceptible plants. *Poaceascoma* accounted for the highest percentage of the unique group of insect-resistant plants topsoil (Ras = 0.08%), and *Ceratobasidiaceae* accounted for the highest proportion (Ras = 0.52%) of the unique group of insect-susceptible plants topsoil. Among the shared fungi, *Nigrospora*, *Neocosmospora* and *Pyrenochaetopsis* were significantly less abundant in the topsoil of insect-resistant plants than that of insect-susceptible plants (*p* < 0.05) (Figures 1, 2).

**Figure 3:**
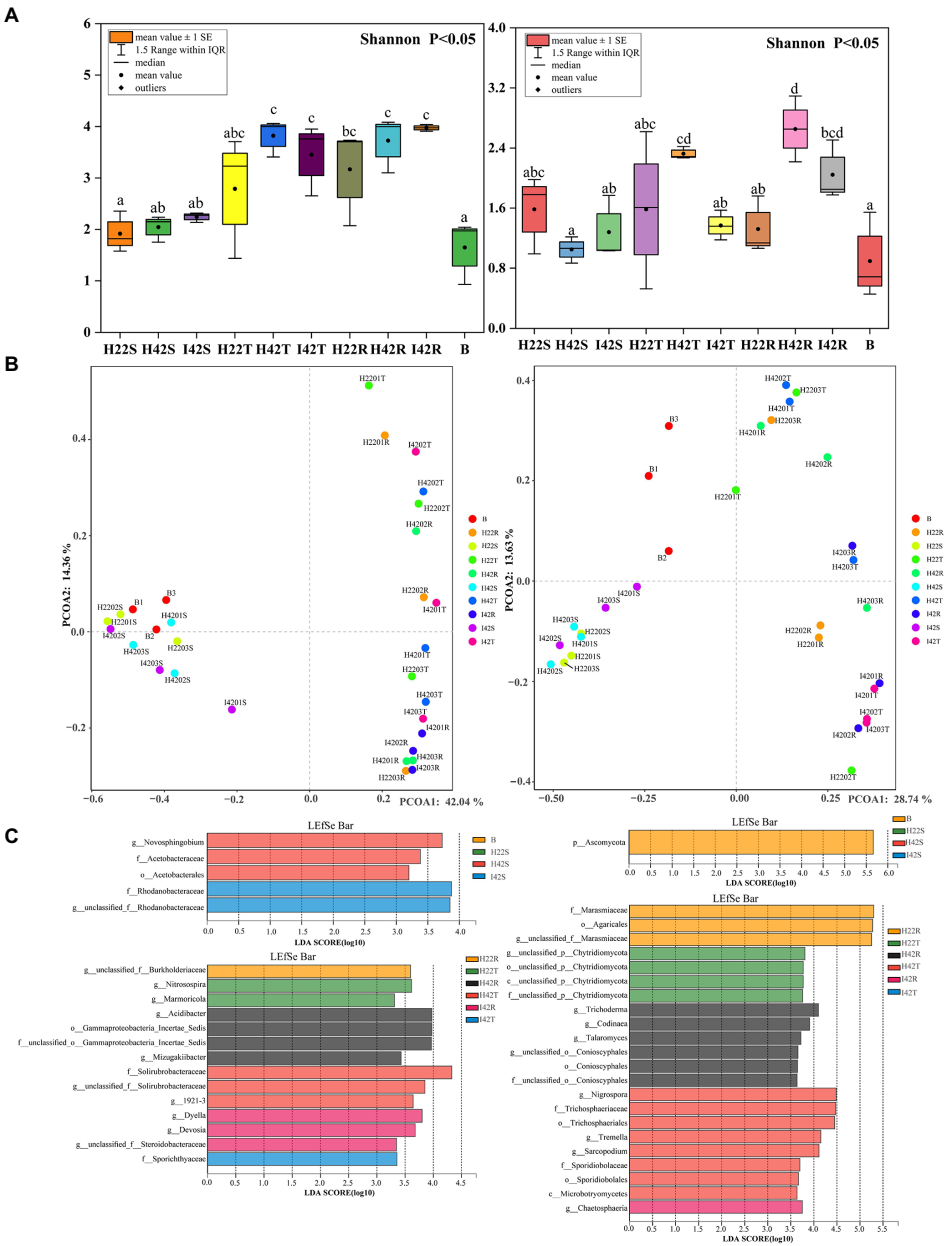
Bacterial (left side) and fungal (right side) differences between groups in alpha, beta diversity variance analysis and differential screening for Microbiome. **(A)** Figure of inter-group differences in alpha diversity of communities characterized at the level of microbiome genera. **(B)** Figure of PCOA of microbiome genus level characterized communities. Based on the obtained table of species diversity characteristics, the Shannon index and Simpson index were selected to calculate the richness and diversity of the community, and the Shannon index was used to plot the comparative differences between groups. PCOA analysis based on bray weighting algorithm. **(C)** (Top left side) Bacterial Microbiome with a significant role in sugarcane stems and herbivores. (top right side) Fungal Microbiome with a significant role in sugarcane stems and herbivores. (Lower left side) Bacterial Microbiome with a significant role in topsoil and rhizosphere soils. (Lower right side) Fungal Microbiome significant in topsoil and rhizosphere soils. LDA discriminant bar charts count the LDA scores obtained by LDA analysis (linear regression analysis) for multiple groups of microbial taxa with significant effects, with larger LDA scores representing greater effects of species abundance on differential effects.

Finally, we found that the microbiome community composition of the inter-rhizosphere soil also differed between insect-resistant and susceptible varieties at the phylum and genus level (one-way ANOVA tests, *p* < 0.05). We obtained a total of 14 phylum-level bacterial microbiome in the inter-rhizosphere of insect-resistant and susceptible plants. The RAs of *Verrucomicrobiota* and *Gemmatimonadota* were significantly higher in inter-rhizosphere soil of resistant plants than in susceptible plants (*p* < 0.05). At the generic level, 109 genera were obtained for the bacterial microbiota, of which 66 were common, 12 unique to inter-rhizosphere soil of insect-resistant plants and 31 unique to inter-rhizosphere soil of insect-susceptible plants. *Ktedonobacteraceae* accounted for the highest proportion of the unique group to inter-rhizosphere soil of insect-resistant plants (Ras = 0.89%). And *Alcaligenaceae* accounted for the highest proportion of microbiota unique to inter-rhizosphere soil of insect-susceptible plants (Ras = 0.35%). *Acidibacter*, *Reyranella*, *Xanthobacteraceae, Acidobacteriales*, *Ellin6067*, *Roseiflexaceae,* and *Oxalobacteraceae* were significantly lower in inter-rhizosphere soil of insect-resistant plants than in that of insect-susceptible plants (*p* < 0.05). Among the fungal microbiota, we obtained six fungal groups: *Glomeromycota*, *Ascomycota, Rozellomycota, Mortierellomycota, Basidiomycota* and *Chytridiomycota*. At the generic level, we obtained a total of 36 genera of fungal, which 17 were common, one unique to inter-rhizosphere soil of insect-resistant plants and 18 unique to inter-rhizosphere soil of insect-susceptible plants. *Glomeraceae* was the most represented microbiome group unique to inter-rhizosphere soil of insect-resistant plants (Ras = 0.07%). *Trechispora, Fusarium,* and *Chaetosphaeria* were significantly lower in inter-rhizosphere soil of resistant plants than in that of susceptible plants (*p* < 0.05) (Figures 2, 3).

### 3.2. Effect of stem borer infestation on the microbiome of soil and sugarcane stem

The microbiome RAs of the stems of the susceptible sugarcane varieties differed significantly at the phylum and genus level before and after the infestation (one-way ANOVA tests, *p* < 0.05). The total number of bacterial and fungal microbiome species in the sugarcane stems increased after borer infestation (Supplementary Figures S3g-i). *Aquabacterium* was the exclusive dominant in post-infested plants (Ras = 11.22%). Among the unique microbiome of pre-infestation plants, *Nigrospora* was the only unique microbiome (Ras = 0.80%) (Figures 1, 2). In contrast, the total number of communities in the soil decreased, except the fungal community species in the rhizosphere soil (Supplementary Figures 3j-l). This indicates that borer infestation can lead to the loss of some bacterial microbiome in top and rhizosphere soil as well as loss of some fungal microbiome in top soil, while other fungal microbiome was added to rhizosphere soil along with the invasion of insects. Interestingly, we found that insect infestation altered the microbial community of the susceptible sugarcane variety and made its microbiome similar with the insect-resistant variety, as evidenced by the convergence of the microbiome at phylum and genus levels, the clustering of samples in the PCOA classification, and the correlation of samples in the hierarchical cluster analysis (Figures 2, 3; Supplementary Figure S5). In addition, the fungal communities in insect-susceptible plants were found to aggregate better with resistant plants after insect damage.

The composition of microbiomes of the topsoil of insect-susceptible plants before and after insect damage differed at the phylum and genus level, but the differences were less than those between insect-resistant and insect-susceptible plants. A total of 13 phylum levels of bacteria were obtained in the topsoil of plants before and after insect damage, which were the same for the insect-resistant plants. At the genus level, we obtained a total of 115 bacterial microbial groups, of which 72 were common to pre and post infestation, 27 unique to pre-infestation plants, and 16 unique to post-infestation plants. At the genus level, we obtained a total of 40 fungal genera, of which 22 were shared, 14 unique to the stripe borer before the damage, and 4 unique to the borer after the damage (Figures 1, 2).

At the generic level, a total of 120 genera were obtained for the bacterial microbiota, 76 before and after stem borer damage, 21 unique to healthy plants and 23 unique to insect-infested plants. Elsterales had the highest proportion (Ras = 0.21%) of microbiota unique to healthy plants, and Caulobacteraceae had the highest proportion (Ras = 0.27%) of microbiota unique to pest-infested plants. The fungal populations in the inter-rhizosphere soil did not show any significant differences before and after the stem borer damage. Xenoacremonium had the highest percentage (Ras = 0.12%) of microflora unique to the inter-rhizosphere soil before stem borer damage, and Echria had the highest percentage (Ras = 0.68%) of microflora unique to the inter-rhizosphere soil after stem borer damage (Figures 1, 2).

### 3.3. Composition and correlation analysis of the microorganisms of the striped borer

We identified a total of four phylum of bacteria from the striped borer, including *Proteobacteria* (Ras = 72.04%), Firmicutes (Ras = 18.54%), *Bacteroidota* (Ras = 8.82%) and *Actinobacteriota* (Ras = 0.58%), and a total of 17 bacterial microbiome at the genus level (Figures 1, 2). We obtained two phyla of fungal microbiome, including *Ascomycota* (Ras = 57.88%) and *Basidiomycota* (Ras = 14.37%), and 10 fungal microbiomes at the genus taxonomic level (Figures 1, 2). We found that the bacterial and fungal microbiome of the striped borer was identical with that of the sugarcane stems of the insect-susceptible plants at the phylum level. At the generic level, the bacterial microbiota of the striped borer and the sugarcane stems damaged by the striped borer shared nine microbiomes, which were not present in the topsoil or inter-root soil (Supplementary Figure S3). The common bacterial microbiome in both the striped borer and the soil were all present in the sugarcane stems. The differences were that the fungal *Candida* in striped borer and the stem was not present in the topsoil or inter-root soil. The striped borer and the topsoil shared one fungal genus, *Schizophyllum*, which was not found in other samples. Four of the fungal genera in the striped borer were present only in the topsoil and rhizosphere, and two were present in all the stem, topsoil and rhizosphere soil. The abundance of the shared bacterial microbiome accounted for 5–10% of the total bacterial microbiome, and the abundance of the shared fungal microbiome accounted for 6–9% of the total fungal microbiome. In general, the bacterial communities of stripe borer were more closely related to the stem, while the fungal communities were more closely related to the topsoil and inter-rhizosphere than to the sugarcane stem (Figure 3).

In the comparison of alpha diversity, the striped borer showed the lowest level of microbiome diversity (one-way ANOVA tests, *p* < 0.05). The microbiome alpha diversity of the stripe borer samples was lower than that of the stems and soil (*p* < 0.05) (Figure 3). The PCOA analysis showed that the bacterial microbiome in the striped borer and sugarcane stems clustered together (PCOA1 = 42.04%, PCOA2 = 14.36%) (Figure 3). This suggested that the bacteria in the striped borer was highly correlated with that of plant stems. The fungal community of all plant stems was clustered into a single area, while that of the striped borer was distributed between the stem and soil (PCOA1 = 28.74%, PCOA2 = 13.63%) (Figure 3). This suggested that the fungal communities of the striped borer were associated with both plant stems and soil.

### 3.4. Correlation of “microbiota-plant–soil-insect” and analysis of differences in functional predictions

To understand microbiome dynamics of ‘plant-insect-soil’ and their role in insect resistance, we integrated the microbiome and soil chemistry of all soil samples and assessed the impact of soil chemistry on the abrupt microbiome. Overall, 19 phyla and 212 bacterial genera were detected in this study, with the top 10 phylum-level bacterial microorganisms accounting for 99.27% of all microbiome. All samples harbored *Aspergillus* (64.99%, the mean of all subgroups), *Actinobacteria* (16.93%) and *Firmicites* (5.68%). We found that these bacterial communities of high abundance were present across the different sugarcane varieties with different percentages (Figure 1). Similarly, a total of 7 phyla and 114 genera were detected in the fungal community. Two fungal micro-organisms, *Tamerella* (49.01%) and *Ascomycetes* (48.67%), accounted for 99.97% of all fungal microbiota (Figure 1). Based on combined bacterial and fungal microbiome, the results showed that the microbiome of plants were likely to be derived from soil, while the microbiome of insects converged with those of plant stems (Figure 3). Worth noting is that the RAs of common bacteria and fungi in sugarcane stem were significantly changed before and after insect infestation (one-way ANOVA tests, *p* < 0.05). For bacteria, the abundance of *Burkholderia* was increased by 921.47%, *Pantoea* by 247.30% and *Enterobacter* by 191.99%. Two main genera of fungi with extreme change was identified, *Saitozyma* with a 1300.00% increase and *Ramichloridium* with a 1204.65% increase. Some microbiomes were unique to plants before or after insect infestation. For example, fungi *Zymoseptoria*, *Sporisorium* and *Candida* (in descending order of abundance) were only detected in infested plants, while *Alcaligenaceae* (bacteria) and *Nigrospora* disappeared after insect damage (Figure 1). The total microbiome community of the soil generally decreased after striped borer damage, with the exception of the fungal community in the rhizosphere soil (Figures 2, 3).

Striped borer infestation affected the RAs of microbiome communities. The alpha diversity of the fungal microbiome was significantly lower (*p* < 0.05) in the rhizosphere soil of insect-resistant varieties in a healthy state than in susceptible plants (Figure 3A). Fungal microorganisms from healthy susceptible plants showed the highest alpha diversity in both surface and inter-root soils, with a decreasing trend after stem borer damage (*p* < 0.05). The microbiome of sugarcane stems showed an increase in alpha diversity of 9.69% (bacteria) and 22.04% (fungi), a decrease of 9.66% (bacteria) and 41.13% (fungi) in the topsoil layer, an increase of 6.62% in bacteria and a decrease of 22.94% in fungi in the inter-rhizosphere after stem borer damage. This suggested that striped borer damage will lead to changes in the microbiome composition and soils, such as the production and loss of some bacterial microbiome and fungal microorganisms, as well as increases and decreases in community abundance.

**Figure 4:**
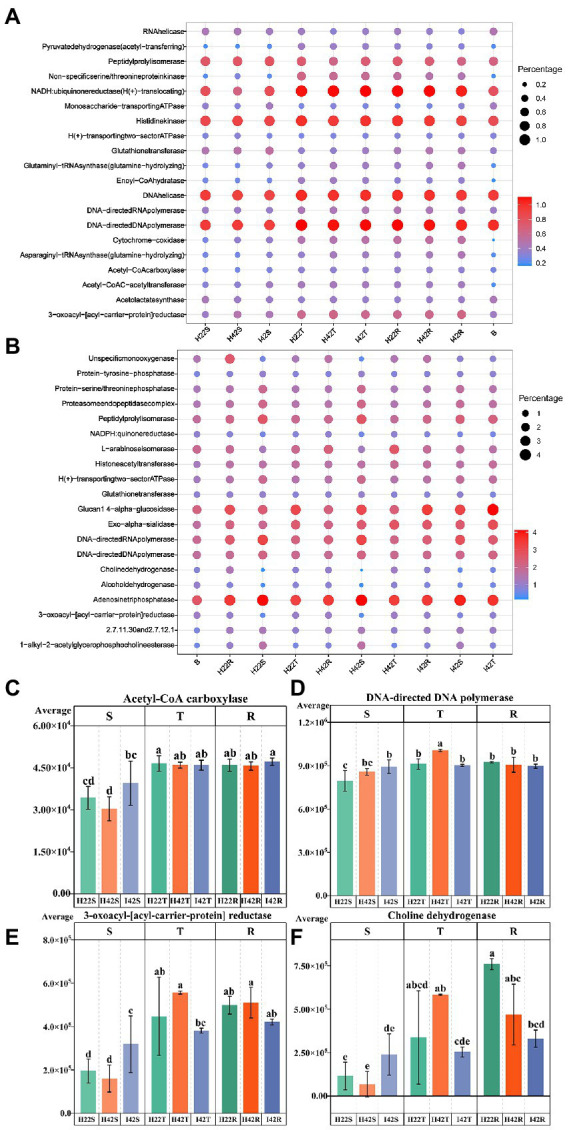
Annotated statistical heatmap of the microbiome function and histogram of the presence of functional differences. The X-axis of the graph represents the mean value of each subgroup, the Y-axis represents the main function obtained from the annotation, the size of the circle represents the abundance of the function and the color represents the correlation coefficient (r). **(A)** Functional annotated bubble diagram of the microbiome of each subgroup of bacteria. **(B)** Functional annotated bubble diagram of the microbiome of each subgroup of fungal. **(C–F)** Microbiome functions that showed the expected significant level of difference in resistance associated with different experimental subgroups. Error bars indicate standard deviation (+/–SD) and letters indicate differences between each subgroup. Differences were significant at the *p* ≤ 0.05 level calculated using the chi-squared test (H).

Similarity analysis and classification based on β-diversity analysis showed that the fungi of soil were closely related to the sugarcane stems with more common microbiome, and the fungal communities of susceptible sugarcane stems were fully contained in the common communities of the topsoil and inter-rhizosphere (Figure 3). We used PICRUSt2 to predict the function of microbiome. Among the first 30 major functional annotations, only one bacterial component with “acetyl coenzyme A carboxylase” showed a significant increase in function in sugarcane stems after insect damage (*p* < 0.05). A total of 21 fungal microbiome functions showed important differences (*p* < 0.05) between sugarcane varieties and before and after insect damage (Figure 4; Supplementary Figure S6). It is noteworthy that the fungal function “DNA-directed DNA polymerase” in the topsoil fraction was significantly lower (*p* < 0.05) in both resistant and post-insect-infested susceptible varieties than in healthy susceptible plants, and the fungal function “3-oxoacyl-[acyl-cattier-protein] reductase” showed a significant increase in the stems before and after insect attack, while it showed a significant decrease in the topsoil before and after insect attack (*p* < 0.05) (Supplementary Figure S6). The results also supported fungi are more sensitive to insect infestation.

**Figure 5:**
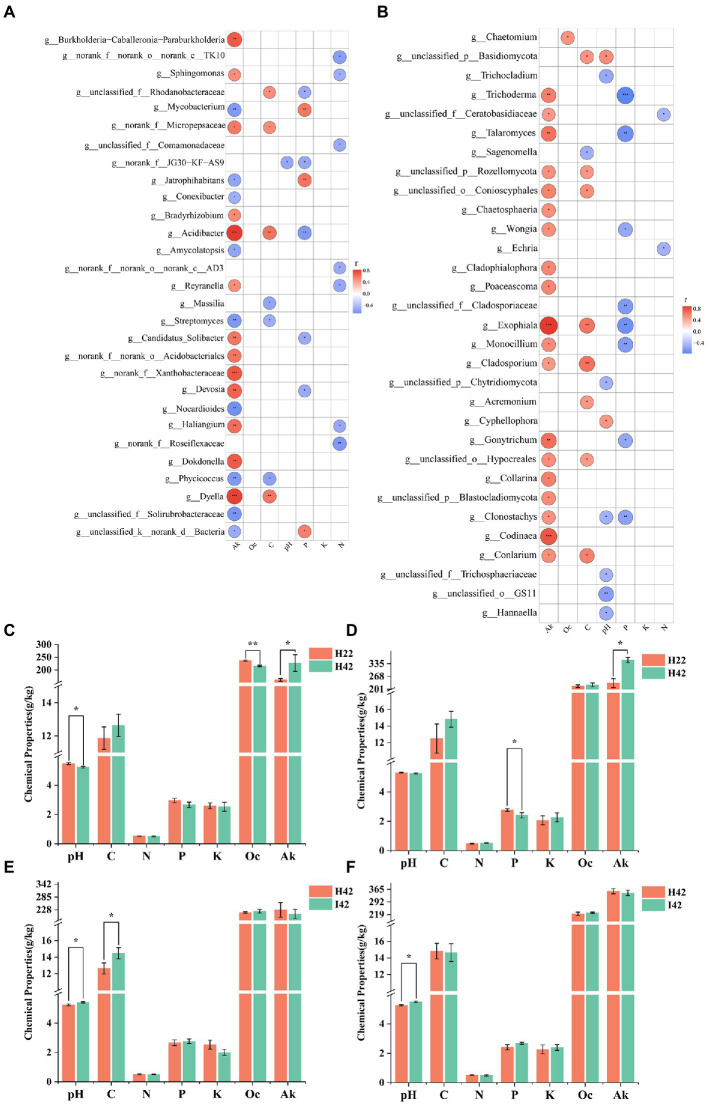
Heatmap of the correlation between the microbiome in soil and soil chemical properties, and statistical histogram of differences in soil chemical properties. Fk for Available Potassium. Oc for Activated organic carbon. C for organic carbon. K for Total potassium. N for Nitrogen. P for Phosphorus. Correlation was calculated by Spearman’s correlation coefficient. Significance markers *p* > =0.05 no marker (white), 0.01 < *p* < 0.05 markers: *, 0.001 < *p* < =0.0 markers: **, *p* < =0.001 markers: ***. **(A)** Heat map of the correlation between microbiome and soil chemistry at the level of bacterial soil genera. **(B)** Heat map of the correlation between microbiome and soil chemistry at the level of fungal soil genera. Positive correlation marker in red, negative correlation marker in blue. **(C)** Soil chemistry between species in the topsoil. **(D)** Soil chemistry between root soil species. **(E)** Soil chemistry before and after insect infestation of the topsoil. **(F)** Soil chemistry before and after insect infestation of the root soil. Soil pH. Soil organic carbon. Soil Available Potassium. Soil total Nitrogen. Soil Total Phosphorus. Total soil potassium. Activated organic carbon. pH determination by potentiometric method, organic carbon determination by potassium dichromate external heating method, total nitrogen determination by Kjeldahl method, total phosphorus determination by NaOH fusion-molybdenum antimony anti-colorimetric method, total potassium determination by NaOH fusion, flame photometric method, easily oxidized organic carbon determination by potassium permanganate oxidation method, available potassium determination by NH4DAc leaching, flame photometric method.

We further measured soil physicochemical properties to analyze the correlation with variability of microbiomes in different sugarcane varieties and/or before and after pest damage (Supplementary Figure S3c). To simply the analysis, we selected the top 50 microbiome groups in terms of abundance level to analyze the correlation with soil chemistry. Soil available potassium showed the highest correlation in both bacteria and fungi, with a total of 21 genera of bacteria significantly correlated (*p* < 0.05), including nine genera with highly significant positive correlation (*p* < 0.01), four genera with significant positive correlation (*p* < 0.05), five genera with highly significant negative correlation *p* < 0.01) and four genera with significant negative correlation (*p* < 0.05) (Figure 5A); 19 genera of fungi were significantly correlated (*p* < 0.05), including 14 genera that were significantly positively correlated (*p* < 0.05) and 5 genera that were highly significantly positively correlated *p* < 0.01) Figure 5B). Readily oxidizable organic carbon was not found to be significantly associated in the bacterial community, and one genus of fungi showed a significant positive association in the fungal community (*p* < 0.05). Organic carbon was significantly correlated (*p* < 0.05) in 7 genera of bacteria in the bacterial community. Two genera were positively correlated (*p* < 0.05), two were highly positively correlated *p* < 0.01) and three were negatively correlated (*p* < 0.05); nine genera of fungi were significantly correlated, including two genera that were highly significantly positively correlated *p* < 0.01), six genera that were positively correlated (*p* < 0.05) and one that was negatively correlated (*p* < 0.05). pH was significantly negatively correlated in the bacterial community in one genus (*p* < 0.05); and seven significant correlations with fungi, including one significant positive correlation (*p* < 0.05), one highly significant negative correlation *p* < 0.01) and five significant negative correlations (*p* < 0.05) for one genus. Total phosphorus was significantly correlated in eight genera in the bacterial community, with two genera significantly positively correlated *p* < 0.01), one genus positively correlated (*p* < 0.05), one highly significantly negatively correlated *p* < 0.01) and four negatively correlated (*p* < 0.05). Total phosphorus was significantly correlated with eight genera in the fungal community, including two highly significant negative correlations *p* < 0.01) and six negative correlations (*p* < 0.05). Total potassium did not show associations with bacteria and fungi. Total nitrogen was significantly associated with 7 genera in the bacterial community, including 1 highly significant negative association *p* < 0.01) and 6 negative associations (*p* < 0.05). A total of 2 genera were significantly negatively correlated (*p* < 0.05) in the fungal community for total nitrogen. A total of 44 genera of bacterial groups and 47 fungal groups were significantly correlated with soil chemical properties (*p* < 0.05) (Figure 5). Soil chemistry was more associated with the soil fungi (Figures 5A,B). Soil available potassium showed the highest correlation among the soil microbiome, while total soil potassium content did not show a correlated microbiome. Organic carbon was more positively correlated with the soil microbiota (*p* < 0.05). pH and total soil phosphorus were more negatively correlated with the soil microbiota (*p* < 0.05). The chaetomium was the only soil microbiota associated with oxidizable organic carbon.

## 4. Discussion

Crop resistance to pests may be highly correlated with the microbiome ecology in which it is found (Hu et al., 2018; Pang et al., 2021). In the last few decades, numerous studies have reported that the soil microorganisms can influence the behavior of terrestrial phytophagous insects by altering the systemic chemistry of the host plant (Borgström et al., 2017; Hannula et al., 2019). The dynamic interactions between aboveground plant and root soil together constitute the microbiome ecology of the crop, and reflect and influence its growth status (Hou et al., 2021; Xiong et al., 2021). In the present study, we verified that the microbiota in the aboveground stems of sugarcane and the striped borer surviving in the stems was predominantly soil-derived, and the fungal microbiome of the striped borer was predominantly from the sugarcane stems and partly from the soil topsoil layer. The fungi showed a stronger response after the insect infestation. Unexpectedly, we found that the overall microbiome of susceptible plants tended to have a similar composition to that of insect-resistant plants after insect damage. The results demonstrated that changes in microbiome were linked to plant defense mechanisms, and plant-insect interactions following insect infestation affected the microbiota of the plant and soil environment.

### 4.1. Dynamic interactions among plant, soil, and microbiomes

**Figure 6:**
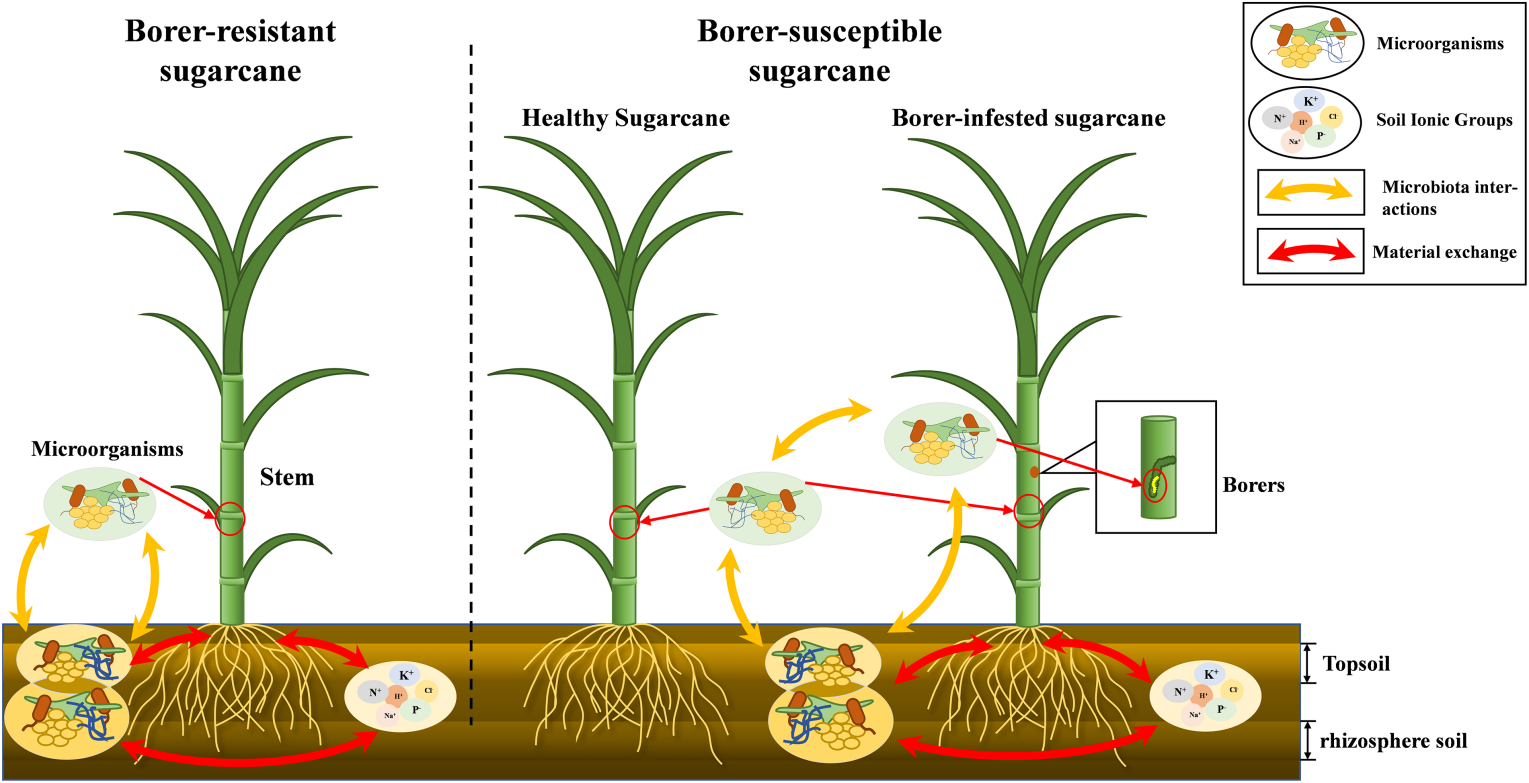
“Microbiota-plant–soil-insect” mechanism diagram. The chemical properties of the soil influence the dynamics of the Microbiome in the soil, and there is a close correlation between the aboveground and belowground soils of plants. There are differences in microbial ecology of different resistant plants. In addition, the invasion of herbivores destabilizes the original Microbiome of plants.

Plant microbiome was dependent on the soil environment, and changes in soil chemistry by plant growth affected the soil microbiota. Many studies have demonstrated the existence of dynamic interactions between plants and soil (Hassani et al., 2018; Choi et al., 2021; Mahmud et al., 2021). We found the microbiome of soil are far more abundant than those of plants, and it was likely that the microbiota of plants was predominantly soil origin. The microbiota structure of the soil can therefore impact the above-ground crop microbiota (Supplementary Figure S2). The differences in the correlation of microbiome between topsoil and rhizosphere soils and plants suggested that soil at different depths contribute differently to the aboveground microbiome community of plants. Bacteria of rhizosphere soil was closely related to the microbiota of the host plant, while fungi of the topsoil were closely related to the microbiota of both plants and insects (Figure 6).

We measured pH, organic carbon, total nitrogen, total phosphorus, total potassium, available potassium and easily oxidisable organic carbon in the soil. The results showed that soil chemistry were changed in sugarcane varieties after a period of plant growth. Plant genotypes have different plant–soil environment interactions, likely resulting in different soil chemical properties (de la Fente Cantó et al., 2020; Semchenko et al., 2022). We analyzed the correlation between soil chemical properties and microbiome communities, and found that *Acidobacteriaceae*, *Sinomonas*, *Poaceascoma*, and *Aspergillus* were present in insect-resistant plants and in post-insect-infested susceptible plants, but not in healthy susceptible plants, which may play an great role in plant resistance to insects (Supplementary Table S1). In a previous report, *Acidobacteriaceae* may determine and limit organic matter degradation (Miyauchi et al., 2020; Shen and Lin, 2021), which is consistent with our findings. *Sinomonas* was reported to have growth-promoting effect. The genus *Poaceascoma* is a new characterized genus in *Scolecospores*, *Lentitheciaceae*, and has not been sufficiently studied. *Aspergillus* is one of the most abundant fungi in the world and in the soil is mainly responsible for decomposing organic matter (Gopal and Gupta, 2016; Bamisile et al., 2018; Getzke et al., 2019).

### 4.2. The establishment of insect microbiome was dependent on plants and soil

Previous studies have pointed out that the microbiome community of insects is mainly derived from the host plant (Frago et al., 2012; Heinen et al., 2018; Malacrinò et al., 2021). Recent studies have suggested that part of the soil effect on the above-ground plant may be due to direct interaction between herbivore and soil microbiota (Hannula et al., 2019; Friman et al., 2021). Yet, the research also points out that the insect microbiota is directly related to the soil, possibly because proceras venosatμm’s life habit in soil during growth. Our study showed that the bacterial microbiota of insects was plant-dominated, while the fungal microbiota was associated with plants and soil. This may be due to the fact that the striped borer larvae feed on the stem and the base of the stem during the seedling stage of sugarcane, where the base of the seedling was close to the soil and the larvae had direct contact with the soil. We hypothesized that the insect’s insect microbiome will also be associated with its activity habits. The dependence of striped borer larvae on stems and soil may vary between periods of striped borer outbreaks and between stages of sugarcane development.

### 4.3. Microorganisms of insect-susceptible sugarcane varieties tended to be similar with insect-resistant sugarcane varieties after pest damage

Sugarcane was grown using the same cultivation practices, but there were significant differences in the composition of soil microorganisms between insect-resistant and insect-susceptible sugarcane varieties, suggesting that plant genotype had a role in determining microbiome. Insect-resistant sugarcane varieties have more unique bacterial and fungal microbiota in their stems than susceptible sugarcane varieties. However, in the surface and rhizosphere soils, susceptible sugarcane varieties had more unique bacterial and fungal microbiota. In our study, the structure of the microbiome of susceptible sugarcane varieties after insect damage converged towards that of insect-resistant varieties (Supplementary Figure S5).

### 4.4. Soil chemistry responded differently to different plant varieties, and before and after pest infestation

In this study, it was found that the inter-root soil pH of resistant varieties was significantly higher than that of sensitive varieties. Previous studies have pointed out that pH is a major factor affecting the soil microbiome community (Crowther et al., 2019). Therefore, soil pH may have an important influence in plant pest resistance and maintaining a suitable soil pH had a positive effect on pest control. Quick-acting potassium was more absorbed in resistant plants, and was highly associated with some bacteria and fungi (Supplementary Figure S4). Previous reports suggest that available potassium appears to play a key role in the functional shaping of fungal potentials, and influencing the viability levels of fungi (Gehring et al., 2014; Christian et al., 2016; Gopal and Gupta, 2016). The change in available potassium may be partially related to changes in the level of individual microorganisms. In our study, available potassium in the topsoil and rhizosphere was significantly lower in insect-resistant plants than in insect-susceptible plants (*p* < 0.05). As the fungal microbiome community structure of insects was partly derived from the soil fungal community, we suggested that low levels of effective potassium in soil might be favorable for insect resistance in sugarcane. Another option was to have a low level of K+ environment in the roots since higher intense uptake of K+ was observed in resistant plants.

When plants are exposed to biotic and abiotic stresses, damage to plant cells result in the loss of some of the K^+^ (Crowther et al., 2019; Li et al., 2019). Thus, resistant plants may store more potassium to ensure resistance to insects (Showler, 2016). Future research will focus on the role of potassium in shaping soil microbiome ecology and establishing the environment for insect-resistant microbiome communities. Readily oxidizable organic carbon, available potassium, total nitrogen and pH are all correlated in insect-resistant microbiome communities, suggesting that fast-acting soil nutrients can influence the structure of microbiome communities in the short term, but that persistent essential soil nutrients can also influence the formation of soil microbiome ecology to some extent (Supplementary Table S2). In previous studies, small changes in soil microbiome composition can determine the interactions between plants and pathogenic bacteria and alter plant health (Shikano et al., 2017; Pineda et al., 2020). Therefore, we suggest that environmental and intrinsic microbiome composition characteristics greatly influence the resistance of aboveground plant parts to insects. Our study showed that the soil–plant relationship is bidirectional, but that the plant microbiome is formed primarily through the soil. In future studies, observing changes in the microbiota at different stages of crop growth will further reveal the influence of crop microbiome ecological effects on plant resistance.

### 4.5. Conclusion

In summary, our findings provided strong and consistent evidence that microbiome is associated with plant resistance to herbivore insects. We found significant differences in the microbiome of resistant and susceptible plants in the stem and underground soil. The microbiome of plants following herbivore attack was similar to that of resistant plants. Meanwhile, we also found that the fungi showed more differences before and after the insect attack. Soil fungal diversity was lower in insect-resistant plants than in insect-sensitive plants, and soil microbiome diversity decreased to a similar level to that of insect-resistant soils after insect damage to insect-sensitive plants. The microbiome of the soil showed the highest diversity in this study, and the microbiome in plant stems was almost entirely derived from the soil. The origin of the microbiota of insects found in our study came mainly from plant stems and partly from the soil. Here we suggest that the origin of insect microbiota may be closely related to their behavior. The chemical properties of the soil showed a significant relevance with the microbiome of the soil, and we found that available potassium showed the highest.

correlation in plant resistance to insects.

## Data availability statement

The datasets presented in this study can be found in online repositories. The names of the repository/repositories and accession number(s) can be found in the article/Supplementary material.

## Author contributions

YZ and XY conceived and designed the experiment. GL, PL, JZ, LS, MZ, and ZJ conducted the empirical work. GL conducted the molecular work, performed the bioinformatics analyses, and analyzed the data. GL, YZ, and XY wrote the manuscript. All authors contributed to the article and approved the submitted version.

## Funding

Funding was provided by the National Natural Science Foundation of China (Grant No. 32100606), Science and Technology Major Project of Guangxi (GK AD21075011), Guangxi Natural Science Foundation (GK AD20297064) and grants from the State Key Laboratory for Conservation and Utilization of Subtropical Agro-Bioresources (SKLCUSA-a202206).

## Conflict of interest

The authors declare that the research was conducted in the absence of any commercial or financial relationships that could be construed as a potential conflict of interest.

## Publisher’s note

All claims expressed in this article are solely those of the authors and do not necessarily represent those of their affiliated organizations, or those of the publisher, the editors and the reviewers. Any product that may be evaluated in this article, or claim that may be made by its manufacturer, is not guaranteed or endorsed by the publisher.

## Footnotes

^1^ https://cloud.majorbio.com

^2^ https://www.cloudtutu.com
