# Supplementary material for: Correlation of microbiomes in “plant-insect-soil” ecosystem
Source: Front Microbiol. 2023 Jan 30;14:1088532. doi: 10.3389/fmicb.2023.1088532 (PMC9922863; doi:10.3389/fmicb.2023.1088532)
Supplement: Supplementary file 1 [file Data_Sheet_1.DOCX]

Supplementary Material

# Supplementary Figures and Tables


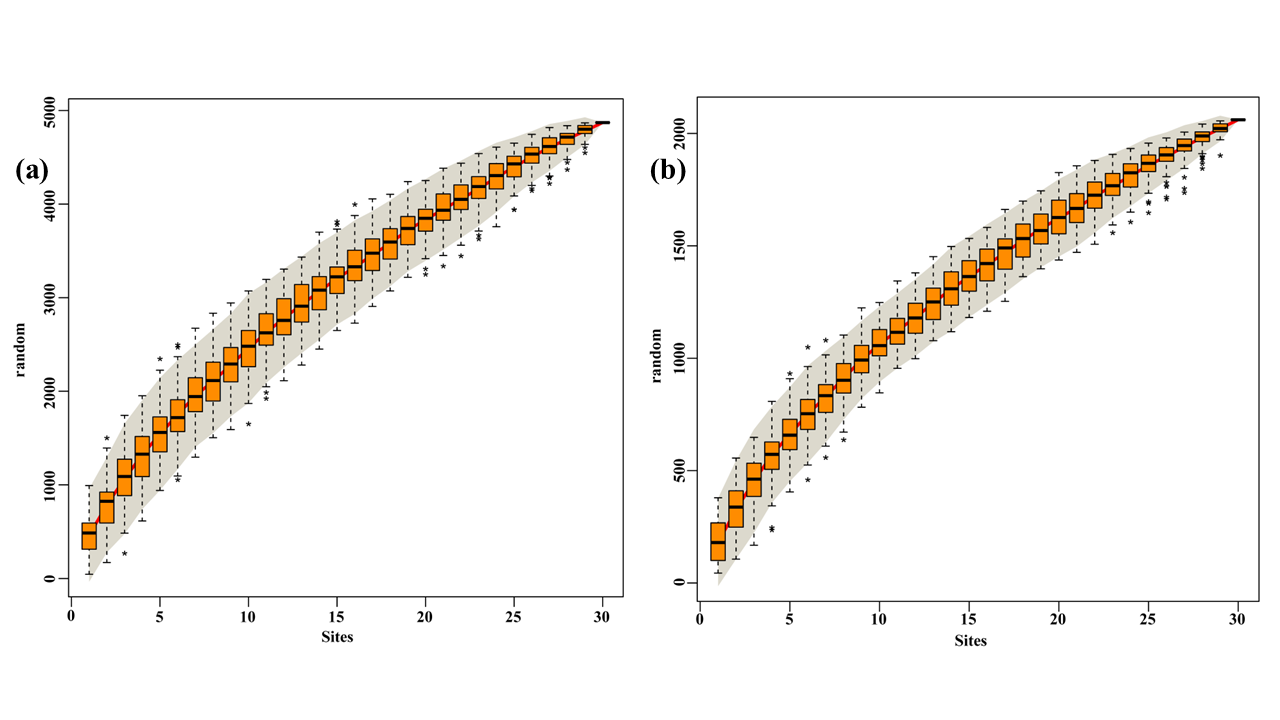


**Fig. S1** Species accumulation curves of bacterial (left side) and fungal (right side). **(a).** Bacterial species accumulation curve. **(b).** Fungal species accumulation curve.


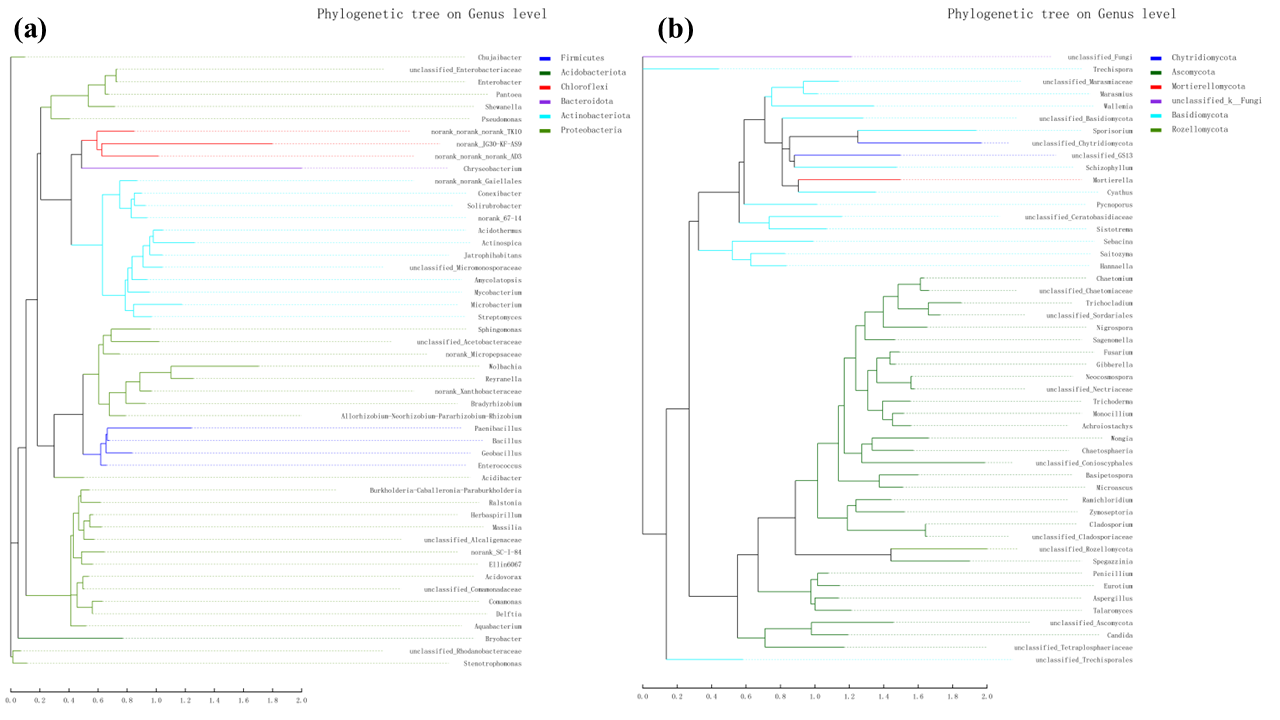


**Fig. S2** Branching diagram of the evolutionary tree of the microbiome in the experiment. **(a).** Map of species evolutionary relationships (bacteria). (**b).** Map of species evolutionary relationships (fungi).


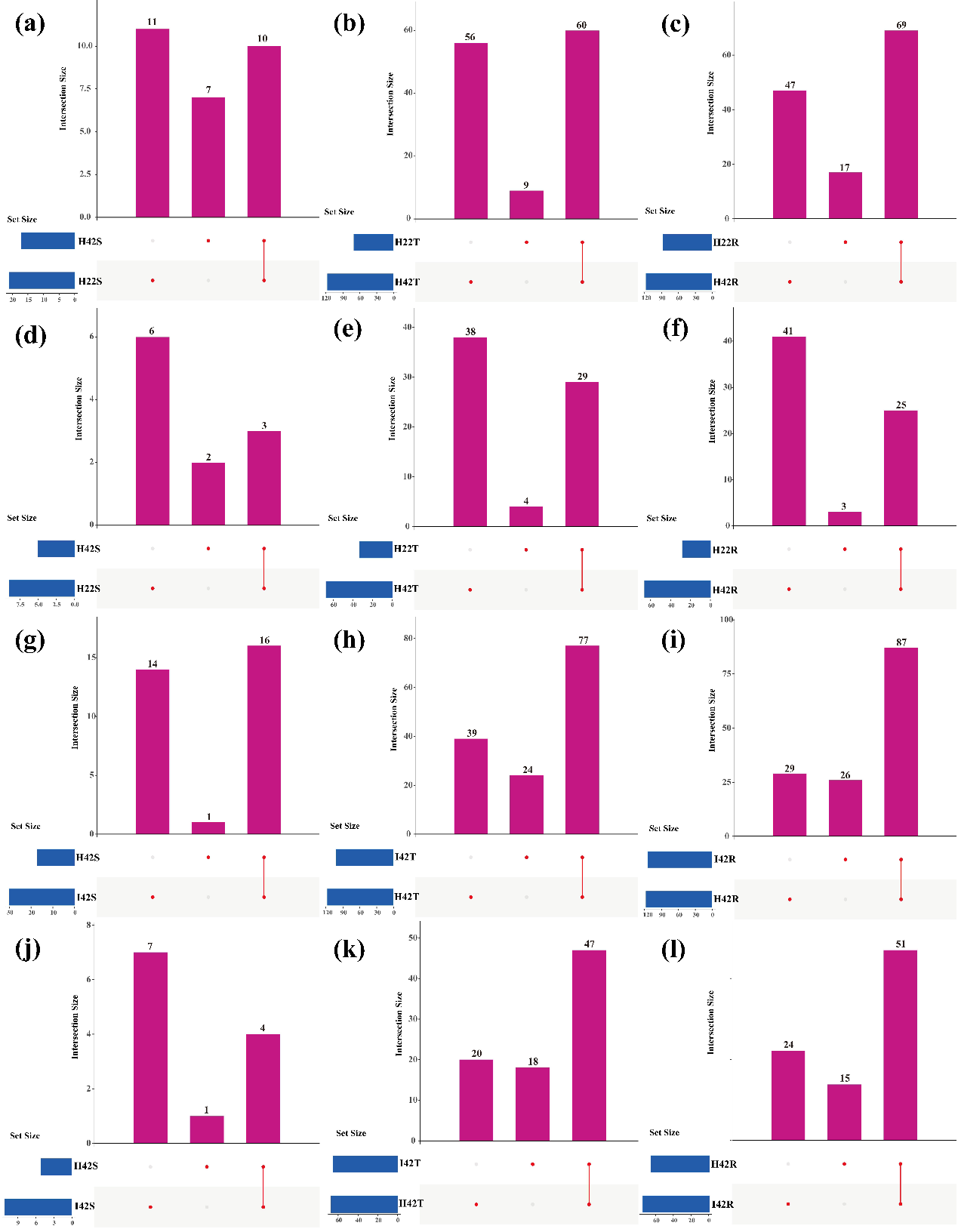


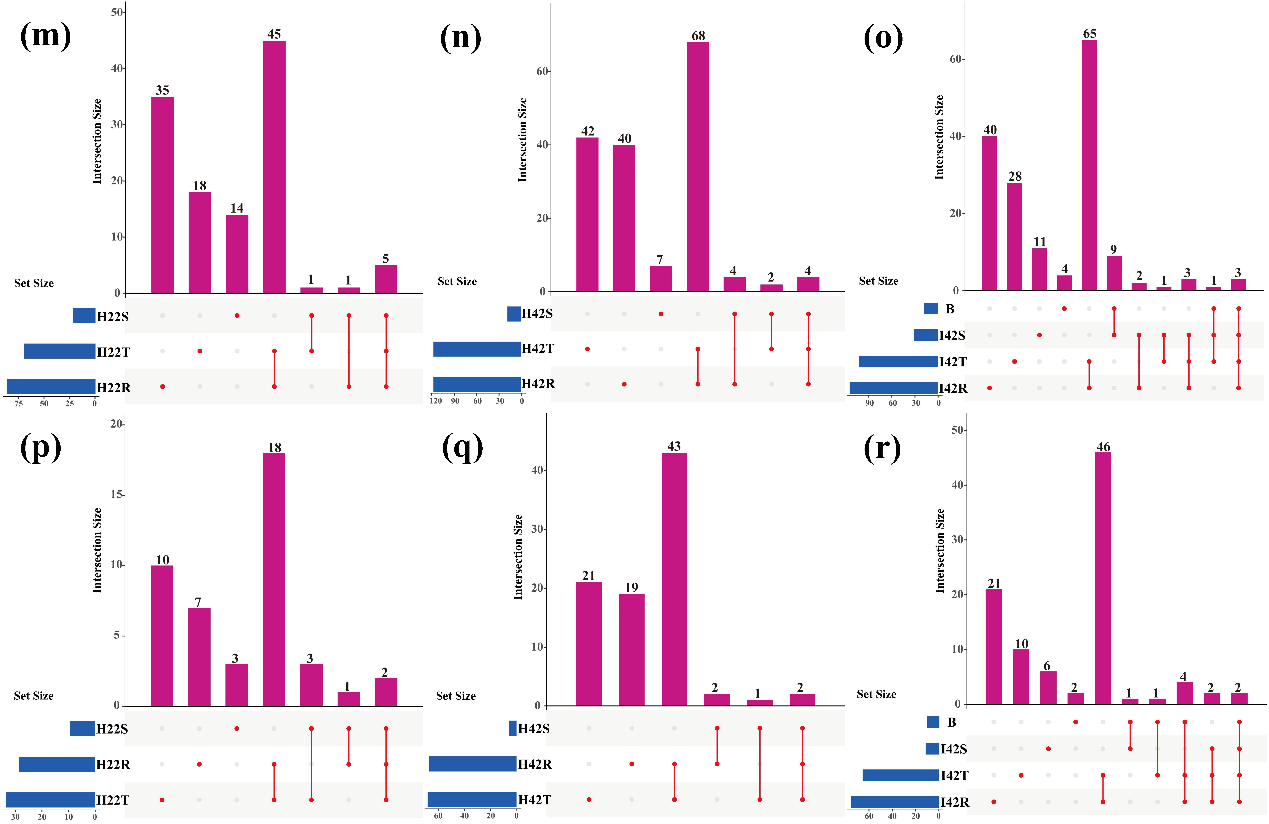


**Fig. S3** Statistics of the common and specific microbiome for the three experimental subgroups. The values are the number of species at the genus level of the Microbiome. **(a).** Differences in stem bacterial communities between varieties; **(b).** Differences in bacterial communities in the topsoil layer between varieties; **(c).** Differences in bacterial communities in the rhizosphere between varieties; **(d).** Differences in stem fungal communities between varieties; **(e).** Differences in fungal communities in the topsoil layer between varieties; **(f).** Differences in fungal communities in the rhizosphere between varieties. Difference analysis between healthy control group and pest test group. The values are the number of species at the genus level of the Microbiome. **(g).** Differences in bacterial communities in the stem of healthy control and pest test groups under the same species; **(h).** Differences in bacterial communities in the topsoil layer of healthy control and pest test groups under the same species; **(i).** Differences in bacterial communities in the root and soil layers of healthy control and pest test groups under the same species; **(j).** Differences in fungal communities in the stem of healthy control and pest test groups under the same species; **(k).** Differences in fungal communities in the topsoil layer of healthy control and pest test groups under the same species **(l).** Differences in fungal communities in the root and soil layers of the healthy control and pest tests under the same species. Correlation analysis of "stem-surface soil-rhizosphere soil" under the same conditions. The values are the number of species at the genus level of the Microbiome. **(m).** Differences in bacterial communities of GT 22 stem-surface soil-interior soil; **(n).** Differences in bacterial communities of GT 42 stem-surface soil-interior soil in healthy control group; **(o).** Differences in bacterial communities of GT 42 stem-surface soil-interior soil in healthy control group in pest test group; **(p).** Differences in fungal communities of GT 22 stem-surface soil-interior soil in healthy control group; **(q).** Differences in fungal communities of GT 42 stem-surface soil-interior soil in healthy control group in healthy control group GT 42 stem-surface soil-rhizosphere soil fungal community differences; **(r).** Pest test group healthy control group GT 42 stem-surface soil-rhizosphere soil fungal community differences.


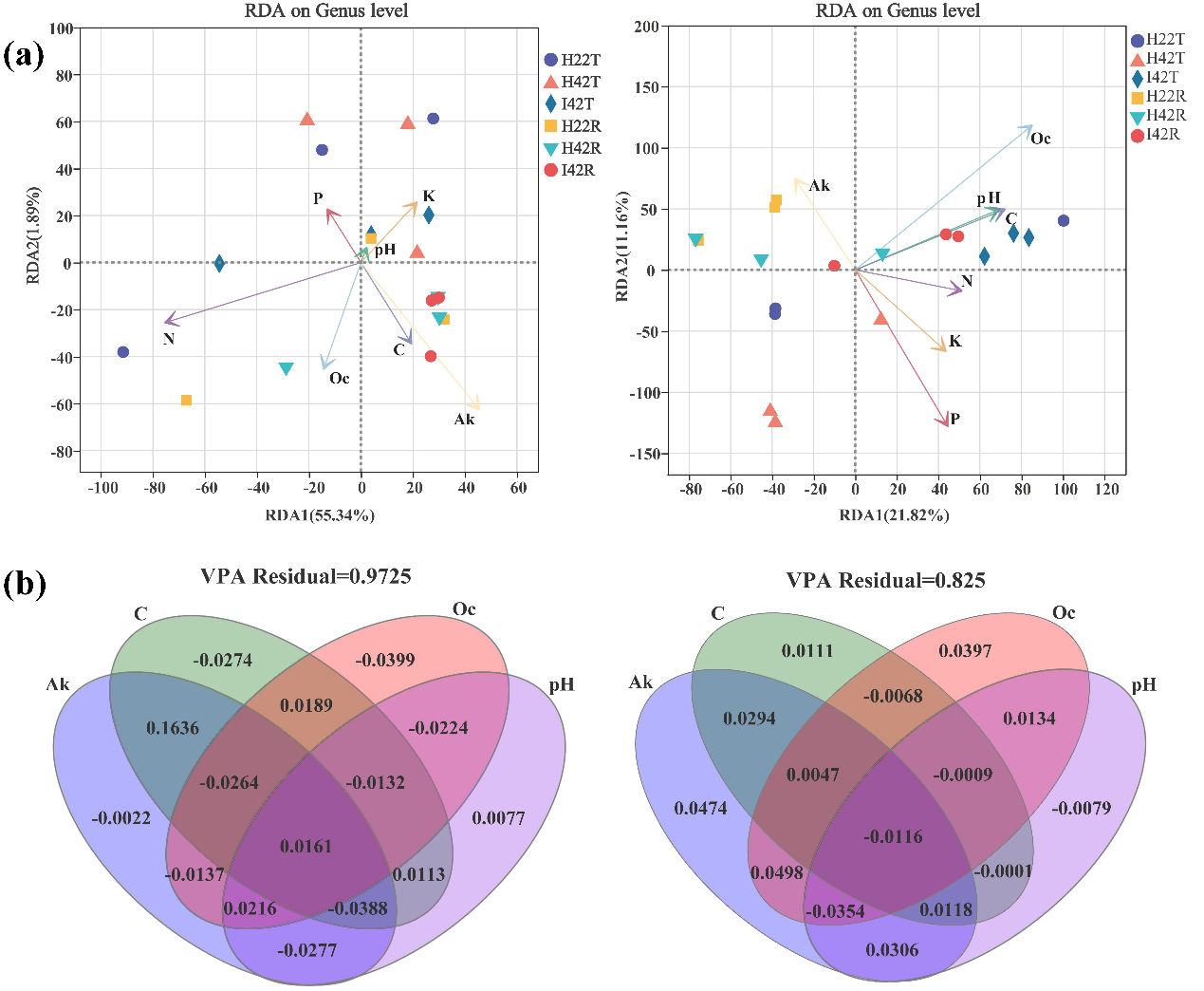


**Fig. S4** The calculated correlation between soil chemical properties and the soil microbiome. **(a)** left side was Bacteria RDA analysis; right side was fungal. **(b).** left side was Bacteria Venn analysis; right side was fungal. The different colored or shaped dots in the graph represent sample groups in different environments or conditions; the light blue inverted triangles represent species; the red arrows represent quantitative environmental factors; the length of the environmental factor arrows can represent the magnitude of the influence of the environmental factor on the distribution of the species data in different groups of samples (the amount of explanation); the angle between the environmental factor arrows represents positive or negative correlation (acute angle: positive correlation; obtuse angle: negative correlation. The angle between the arrows of the environmental factors represents positive and negative correlation (acute angle: positive correlation; obtuse angle: negative correlation; right angle: no correlation).


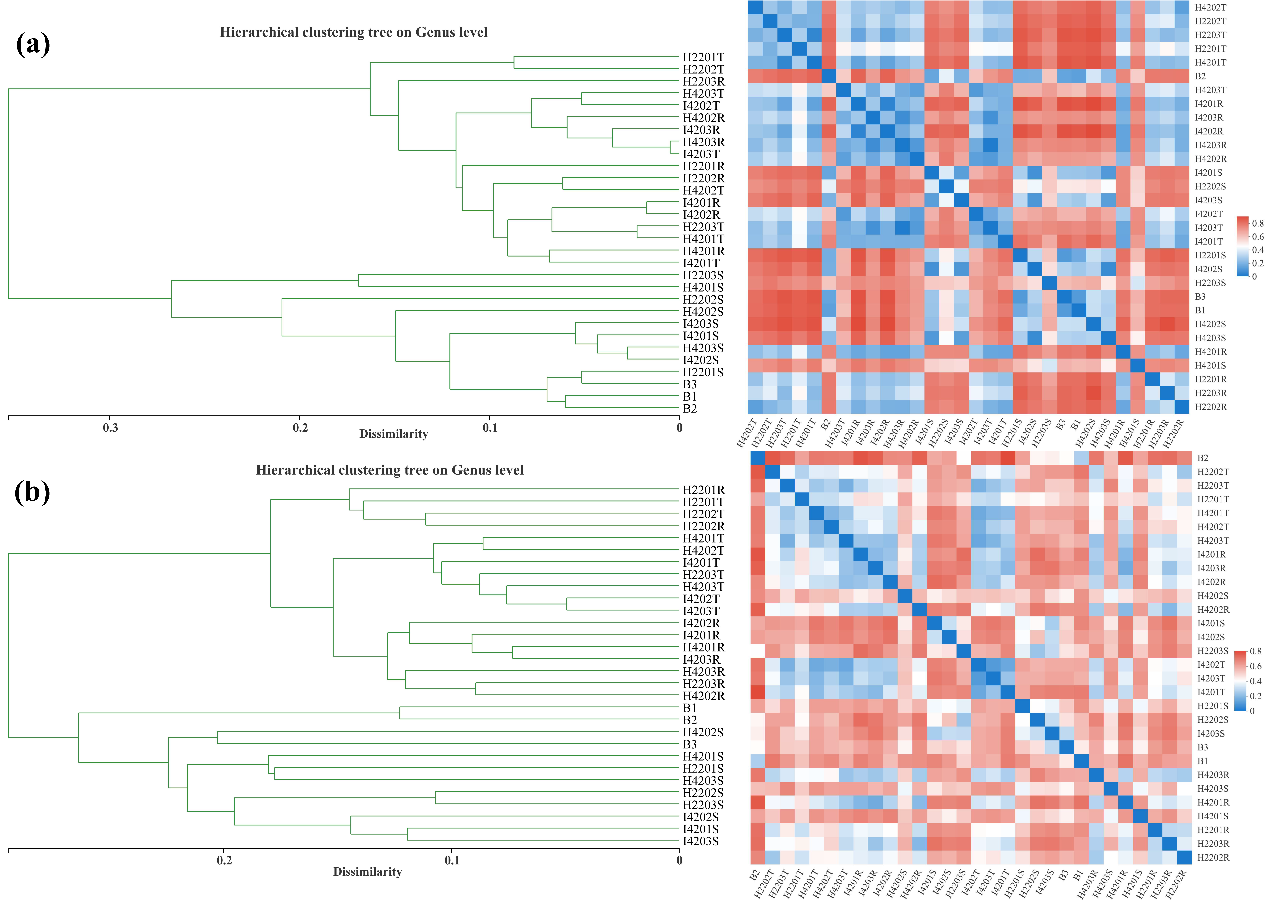


**Fig. S5** Hierarchical clustering plots and sample correlation heatmaps.. **(a).** Bacteria. **(b).** fungal. Hierarchical clustering analysis was carried out based on the beta diversity distance matrix, and a tree structure was constructed using the UPGMA algorithm, with the length of the branches representing the distance between samples. Both the X and Y axes of the heat map are samples, and the distances between samples are represented by different colour gradients (the values represented by the colour gradients are shown on the right-hand side of the figure).


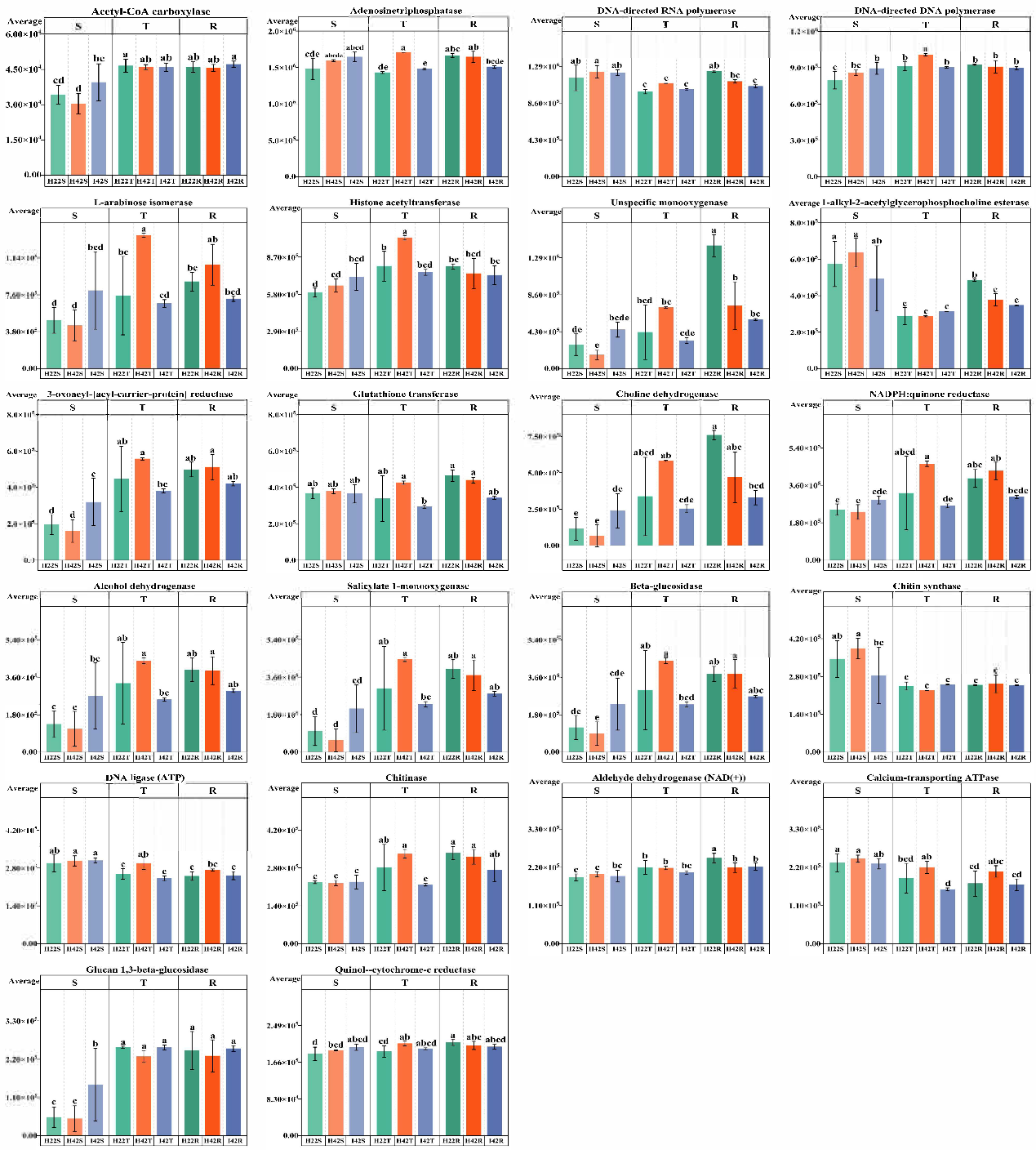


**Fig. S6** Histogram group of functional differences in microbiome. Microbiome functions that showed the expected significant level of difference in resistance associated with different experimental subgroups. Error bars indicate standard deviation (+/-SD) and letters indicate differences between each subgroup. Differences were significant at the p≤0.05 level calculated using the chi-squared test (H).

**Table S1**

**Number of bacterial communities(genus) exhibiting similar pattern in borer-resistant sugarcane and infested susceptible sugarcane**

| **Genus** | **H22** | **H42** | **I42** | **Sampling location** |
| --- | --- | --- | --- | --- |
| g__unclassified_f__Rhodanobacteraceae | 43 | 0 | 676 | **stem** |
| g__unclassified_f__Acidobacteriaceae_Subgroup_1 | 48 | 0 | 42 | **topsoil (5-15cm)** |
| g__Ramlibacter | 98 | 0 | 128 |  |
| g__norank_f__Sporichthyaceae | 86 | 0 | 100 |  |
| g__unclassified_f__Rhizobiaceae | 168 | 0 | 109 | **rhizosphere soil (25-35cm)** |
| g__unclassified_c__Actinobacteria | 196 | 0 | 81 |  |
| g__norank_f__TRA3-20 | 67 | 0 | 75 |  |
| g__Sinomonas | 45 | 0 | 55 |  |
| g__unclassified_c__Alphaproteobacteria | 43 | 0 | 21 |  |

**Number of fungal communities(genus) exhibiting similar pattern in borer-resistant sugarcane and infested susceptible sugarcane**

| **Genus** | **H22S** | **H42S** | **I42S** | **Sampling location** |
| --- | --- | --- | --- | --- |
| g__Trechispora | 718 | 0 | 19 | **stem** |
| g__Candida | 138 | 0 | 703 |  |
| g__Poaceascoma | 131 | 0 | 30 | **topsoil (5-15cm)** |
| g__Aspergillus | 89 | 0 | 8 | **rhizosphere soil (25-35cm)** |
| g__Acremoniopsis | 24 | 0 | 37 |  |

**Table S2**

**Bacterial Envfit Environmental Factor Table**

| **Sample** | **RDA1** | **RDA2** | **r2** | **p_value** |
| --- | --- | --- | --- | --- |
| pH | 0.0895 | 0.996 | 0.0018 | 0.987 |
| C | 0.5747 | -0.8183 | 0.1517 | 0.285 |
| N | -0.9956 | -0.0938 | 0.4347 | 0.011 |
| P | -0.5767 | 0.817 | 0.0635 | 0.623 |
| K | 0.5815 | 0.8136 | 0.0652 | 0.595 |
| Oc | -0.0565 | -0.9984 | 0.1555 | 0.279 |
| Ak | 0.6304 | -0.7763 | 0.6184 | 0.001 |

**Fungal Envfit Environmental Factor Table**

| **Sample** | **RDA1** | **RDA2** | **r2** | **p_value** |
| --- | --- | --- | --- | --- |
| pH | 0.8261 | 0.5635 | 0.0682 | 0.594 |
| C | 0.8446 | 0.5354 | 0.0721 | 0.563 |
| N | 0.8334 | -0.5527 | 0.0439 | 0.692 |
| P | 0.4627 | -0.8865 | 0.3212 | 0.04 |
| K | 0.5742 | -0.8187 | 0.1132 | 0.385 |
| Oc | 0.3729 | 0.9279 | 0.2199 | 0.147 |
| Ak | -0.4795 | 0.8775 | 0.1107 | 0.429 |

**Table S3 Primer information**

| **Sequencing Area** | **Primer Names** | **Primer Sequences** |
| --- | --- | --- |
| 799F_1193R | 799F | AACMGGATTAGATACCCKG |
|  | 1193R | ACGTCATCCCCACCTTCC |
| ITS1F_ITS2R | ITS1F | CTTGGTCATTTAGAGGAAGTAA |
|  | ITS2R | GCTGCGTTCTTCATCGATGC |

**Table S4**

| **PCR amplification conditions (bacteria)** | | | | | | | | | | |
| --- | --- | --- | --- | --- | --- | --- | --- | --- | --- | --- |
| The PCR formal test was performed using TransGen AP221-02: TransStart Fastpfu DNA Polymerase, 20 μl reaction system. | | | | | | | | | | |
|  |  |  |  |  |  |  |  |  |  |  |
| 5×FastPfu Buffer ............................... 4 μl | | | | | | | | | | |
| 2.5 mM dNTPs ................................... 2 μl | | | | | | | | | | |
| Forward Primer(5 μM) ................... 0.8 μl | | | | | | | | | | |
| Reverse Primer(5 μM) …............... 0.8 μl | | | | | | | | | | |
| FastPfu Polymerase ........................ 0.4 μl | | | | | | | | | | |
| BSA .................................................. 0.2 μl | | | | | | | | | | |
| Template DNA ................................. 10 ng | | | | | | | | | | |
|  |  |  |  |  |  |  |  |  |  |  |
| Supplement ddH2O to ..................... 20 μl | | | | | | | | | | |
| PCR instrument: ABI GeneAmp® Model 9700 | | | | | | | | | | |
| PCR reaction parameters. | | | | | | | | | | |
| a. 1× (3 minutes at 95°C) | | | | | | | | | | |
| b. Number of cycles × (30 seconds at 95°C；30 seconds at refractory temperature °C；  45 seconds at 72°C) | | | | | | | | | | |
| c. 10 minutes at 72°C，10°C until halted by user | | | | | | | | | | |
|  |  |  |  |  |  |  |  |  |  |  |
| Second round amplification | | | | | | | | | | |
|  |  |  |  |  |  |  |  |  |  |  |
| One round of primers 799F-1392R, annealed at 55°C, cycle number 27cycles | | | | | | | | | | |
|  |  |  |  |  |  |  |  |  |  |  |
| Second round primers 799F-1193R, annealed at 55°C, cycle number 13cycles | | | | | | | | | | |

| **PCR amplification conditions (Fungi)** | | | | | | | | | | |
| --- | --- | --- | --- | --- | --- | --- | --- | --- | --- | --- |
| The PCR formal test was performed using TaKaRa rTaq DNA Polymerase, 20 μl reaction system | | | | | | | | |  |  |
|  |  |  |  |  |  |  |  |  |  |  |
| 10× Buffer .......................................... 2 μl | | | | | | | | | | |
| 2.5 mM dNTPs ................................... 2 μl | | | | | | | | | | |
| Forward Primer(5 μM) ................... 0.8 μl | | | | | | | | | | |
| Reverse Primer(5 μM) …............... 0.8 μl | | | | | | | | | | |
| rTaq Polymerase ............................. 0.2 μl | | | | | | | | | | |
| BSA .................................................. 0.2 μl | | | | | | | | | | |
| Template DNA ................................. 10 ng | | | | | | | | | | |
|  |  |  |  |  |  |  |  |  |  |  |
| Supplement ddH2O to ..................... 20 μl | | | | | | | | | | |
| PCR instrument: ABI GeneAmp® Model 9700 | | | | | | | | | | |
| PCR reaction parameters. | | | | | | | | | | |
| a. 1× (3 minutes at 95°C) | | | | | | | | | | |
| b. Number of cycles × (30 seconds at 95°C；30 seconds at refractory temperature °C；45 seconds at 72°C) | | | | | | | | | | |
| c. 10 minutes at 72°C，10°C until halted by user | | | | | | | | | | |
